# Development of a Novel Technique for the Measurement of Neuromuscular Junction Functionality in Isotonic Conditions

**DOI:** 10.1007/s12195-022-00721-3

**Published:** 2022-04-07

**Authors:** Flavia Forconi, Ludovica Apa, Simona Pisu, Irene Casola, Antonio Musarò, Emanuele Rizzuto, Zaccaria Del Prete

**Affiliations:** 1 DAHFMO – Unit of Histology and Medical Embryology, Sapienza University of Rome, Via Antonio Scarpa 16, Rome, Italy; 2 Department of Mechanical and Aerospace Engineering, Sapienza University of Rome, Via Eudossiana 18, 00184 Rome, Italy

**Keywords:** Membrane stimulation, Nerve stimulation, *In-situ* functional measurements, Isotonic fatigue, Experimental optimal force, Maximum power, Neurotransmission failure, Isotonic neurotransmission failure

## Abstract

**Introduction:**

The neuromuscular junction (NMJ) is a chemical synapse responsible for converting electrical pulses generated by the motor neuron into electrical activity in muscle fibers, and is severely impaired in various diseases, such as Amyotrophic Lateral Sclerosis (ALS). Here, we proposed a novel technique to measure, for the first time, NMJ functionality in isotonic conditions, which better reflect muscle physiological activity.

**Methods:**

We employed the *in-situ* testing technique, studied a proper placing of two pairs of wire electrodes for nerve and muscle stimulation, developed an extensive testing protocol, and proposed a novel parameter, the Isotonic Neurotransmission Failure (INF), to properly capture the impairments in neurotransmission during isotonic fatigue. We employed wild-type mice to assess the feasibility of the proposed technique, and the ALS model SOD1^G93A^ mice to demonstrate the validity of the INF*.*

**Results:**

Results confirmed the measurement accuracy in term of average value and coefficient of variation of the parameters measured through nerve stimulation in comparison with the corresponding values obtained for membrane stimulation. The INF values computed for the SOD1^G93A^ tibialis anterior muscles pointed out an impairment of ALS mice during the isotonic fatigue test, whereas, as expected, their resistance to fatigue was higher.

**Conclusions:**

In this work we devised a novel technique and a new parameter for a deep assessment of NMJ functionality in isotonic conditions, including fatigue, which is the most crucial condition for the neuronal signal transmission. This technique may be applied to other animal models, to unravel the mechanisms behind muscle-nerve impairments in other neurodegenerative pathologies.

## Introduction

The neuromuscular junction (NMJ) is a specialized chemical synapse with an important role in transmitting and amplifying information from spinal motor neuron to skeletal muscle.^20^ NMJ functionality can be measured by comparing the contractile response of the muscle elicited by direct stimulation, occurring when electrical pulses are delivered on muscle membrane, and indirect stimulation, obtained with a punctual stimulation of the nerve.^1,6^ Since the direct stimulation completely bypasses the neurotransmission signaling, any differences in the two responses can be attributed to alterations in the NMJ. Changes in contractile kinetics, maximum and specific force may be measured. However, the most interesting evaluation of NMJ functionality concerns the capability of transmitting the neuronal signal when subjected to fatigue. Indeed, when the muscle is repeatedly stimulated to contract, it develops fatigue and the synaptic transmission is impaired.^2^ Different studies have extensively investigated the NMJ fatigue of muscle-nerve preparations in isometric condition, i.e. the muscle is kept fixed at a constant length.^7,8,17,19^ For example, Fogarty et al. investigated the neuromuscular transmission failure of an *ex-vivo* tibialis anterior and of a diaphragm muscle in a rodent model of hypertonia and in aged rats^7^,^8^. They showed that the early-onset hypertonia is strongly associated with impaired neuromuscular transmission, and revealed an altered neuromuscular transmission in diaphragm muscles of aged rats at higher frequency, which is involved in muscle sarcopenia in old rats. On the other hand, Personius and Sawyer^17^ investigated the extent of neurotransmission failure in the diaphragm of adult dystrophic mdx mice. They proposed an isometric fatigue protocol in which a single direct stimulation was followed by 14 indirect stimulations, with the aim of stressing the NMJ, exposing it at higher physiological firing frequencies. The fatigue of the synaptic transmission can be evaluated through two specific parameters: the Neurotransmission Failure (NF), which expresses the difference in the force decrease obtained through the indirect and direct stimulations normalized to that obtained through the direct stimulation during the first contraction,^1^ and the Intratrain Fatigue (IF), which represents the force decrease within a single pulse train of stimulation.^27^ Similarly, in a previous study, we measured NMJ functionality in Soleus and diaphragm muscles of SOD1^G93A^ Amyotrophic Lateral Sclerosis mouse model, also through the measurement of NF and IF parameters.^22^ Although all these previous studies contributed to obtain a more comprehensive understanding of NMJ functionality in different pathologies, they were all carried out in isometric conditions. However, the situation that better reflects the *in-vivo* skeletal muscle dynamic activity is the isotonic one, in which the muscle is allowed shortening, and, to the best of the authors’ knowledge, this approach has never been attempted to study NMJ functionality. On the contrary, different studies^3,4,23,25^ focused on the investigation of isotonic fatigue only for direct stimulation on several muscle types. It is also crucial to remark that in all these studies approaching the measurement of muscle isotonic fatigue, the specimen to be tested was repeatedly stimulated to shorten against a load equal to one-third of its maximal force, known as reference optimal force.^14,16^ This value was chosen as the best representative of the force at which the muscle produced its maximum power.^14,25,28^ However, when testing muscle types from different animal models, the reference optimal force may significantly differ from the force at which the muscle really generates its maximum power. Consequently, significant errors may be introduced in all the parameters measured during isotonic fatigue. Based on this assumption, in a previous work,^9^ we proposed to measure the resistance to isotonic fatigue by subjecting each muscle to its experimental optimal force, i.e. the force at which the muscle really developed the maximum power, computed through a real-time measurement of the power generation during the application of the after-load technique.

Within all this context, here we propose a novel experimental technique, together with an extensive testing protocol, for the measurement of neuromuscular junction functionality of murine animal models in isotonic conditions. To do this, we applied the *in-situ* methodology to study NMJ functionality of wild-type (WT) tibialis anterior (TA) muscles, by stimulating them directly on the membrane and indirectly through the sciatic nerve. Indeed, since in a healthy animal no NMJ defects are expected, the results obtained through the nerve stimulation must be in accordance with that obtained from direct membrane stimulation, thus providing an indirect validation of the proposed technique. Moreover, during the isotonic fatigue we yielded the muscle lifting an external load equal to the experimental optimal force, computed separately for both the direct and indirect stimulation. To extensively characterize the fatigue behavior in isotonic conditions we also proposed a new parameter, the *Isotonic Neurotransmission Failure* (*INF*), which computes the difference in the shortenings occurred when stimulating the muscle through the nerve and the membrane, normalized to that obtained when stimulating the muscle on the membrane during the first pulse train.

It is important to remark that an altered NMJ functionality can be found in several pathologies, such as aging, acute denervation, Duchenne Muscular Dystrophy (DMD) and Amyotrophic Lateral sclerosis (ALS).^5,10,13,24^ In particular, ALS is the most frequent neurodegenerative disease where the progressive failure of the neuromuscular system results in weakness and atrophy of the limb muscle, gradual paralysis and death from respiratory failure.^5,21^ Among all the animal models used to study ALS, SOD1^G93A^ mouse is one of the most employed^12^ and it is based on the expression of the human SOD1 protein containing the G93A mutation.^12,15^ In view of this, after having taken advantage of control wild-type mice to assess the feasibility of the proposed technique, we proceeded with a proof of concept of the *INF*, computing it for SOD1^G93A^ TA muscles at the end-stage of the disease. Indeed, at this age an alteration in the communication between muscle and nerve has been already reported for this animal model in isometric conditions and could be therefore expected also for the isotonic ones. Results showed a significant difference in the *INF* between SOD1^G93A^ and WT mice, confirming the capability of this parameter of assessing impairments in the synaptic transmission during isotonic fatigue test.

## Materials and Methods

### Experimental Procedure

All experiments were conducted within the animal welfare regulations and guidelines of the Italian National Law D.L. 04/03/2014, n.26, about the use of animals for research. Neuromuscular junction (NMJ) functionality has been evaluated through the *in-situ* methodology by comparing muscle contractile response elicited by direct stimulation on tibialis anterior (TA) muscle membrane and indirect one through the sciatic nerve. Ten 5-month-old wild-type (WT) C57BL/6 mice were employed in this study to assess the precise sequences and resting times of the testing protocol for the measurement of isometric and isotonic parameters of muscle and NMJ functionality. Eight 5-month-old WT mice were then used with the final procedure to compute all the parameters proposed in the experimental protocol, as a validation of the proposed technique, and one tibialis anterior - sciatic nerve preparation was tested for each animal. Five SOD1^G93A^ mice were then used to compute the *Isotonic Neurotransmission Failure*.

**Figure 1:**
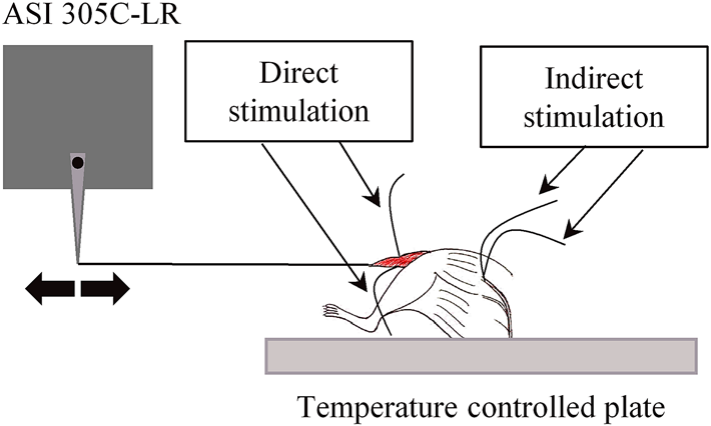
Schematic of the electrodes positioning for direct and indirect stimulations. Tibialis anterior muscle is in red, sciatic nerve is grey.

Before the beginning of the experiment, the mouse to be tested was anesthetized with an intraperitoneal injection of Ketamina Cloridrato (Ketalar) and, during the experiment, a supplemental dose was given if necessary. The skin of the left hind limb muscles was removed, and the TA was identified. Its tendon was cut a few millimeters far from the end of the muscle, taking care not to include the tendon of Extensor Digitorum Longus muscle in the surgical isolation. The surrounding muscles were removed to expose the sciatic nerve. The mouse was then placed on a temperature-controlled plate adjusted to maintain the body temperature at 37 ± 1°C, the hind limb was inserted in a clamp to immobilize it as much as possible, and the foot was scotch-taped to the platform. During the test, the exposed tissues were kept moist by periodic application of mineral oil.^19^ The TA tendon was tied with a 0.16 mm diameter nylon wire slip knot as close to the muscle attachment as possible and connected to the level-arm of a dual-mode actuator/transducer system (305C-LR, Aurora Scientific), as shown in Fig. 1. In particular, the level-arm could be controlled either in force or in position mode, allowing to continuously switch between isometric and isotonic stimulation.

Muscle contractility was elicited by two pairs of stranded stainless steel wire electrodes (AS632 Cooner Wire): for indirect stimulation the electrodes were placed close to the sciatic nerve, while for the direct one they were inserted laterally just under TA muscle surface, as shown in Fig. 1. Electrical square pulses of about 7 mA for nerve stimulation and about 5 mA for muscle one were generated by two electrical pulse stimulators (701C Aurora Scientific) with a width of 1 ms.^19^

The actuator/transducer and the pulse stimulators were controlled by a custom-made software developed in LabVIEW 2019 (National Instruments) using a data acquisition board (PCIe-6363X, National Instruments). This software allowed for choosing all the stimulation parameters, as the experimental protocol, i.e. the number and type of stimulations and the resting times for the entire testing protocol, the force offset value and the percentage values of tetanic force for the after-load technique, while simultaneously acquiring muscle shortening, force, time derivative of force, shortening velocity and pulse sequence for post-processing.

A digital oscilloscope (Tektronix DPO2014B) was included in the experimental set-up for the real-time visualization of force and length. The optimal initial length was obtained by subjecting the muscle to a series of single pulses (usually 4 to 6) to find out the maximum twitch force ^22^.

### Experimental Protocol

**Figure 2:**
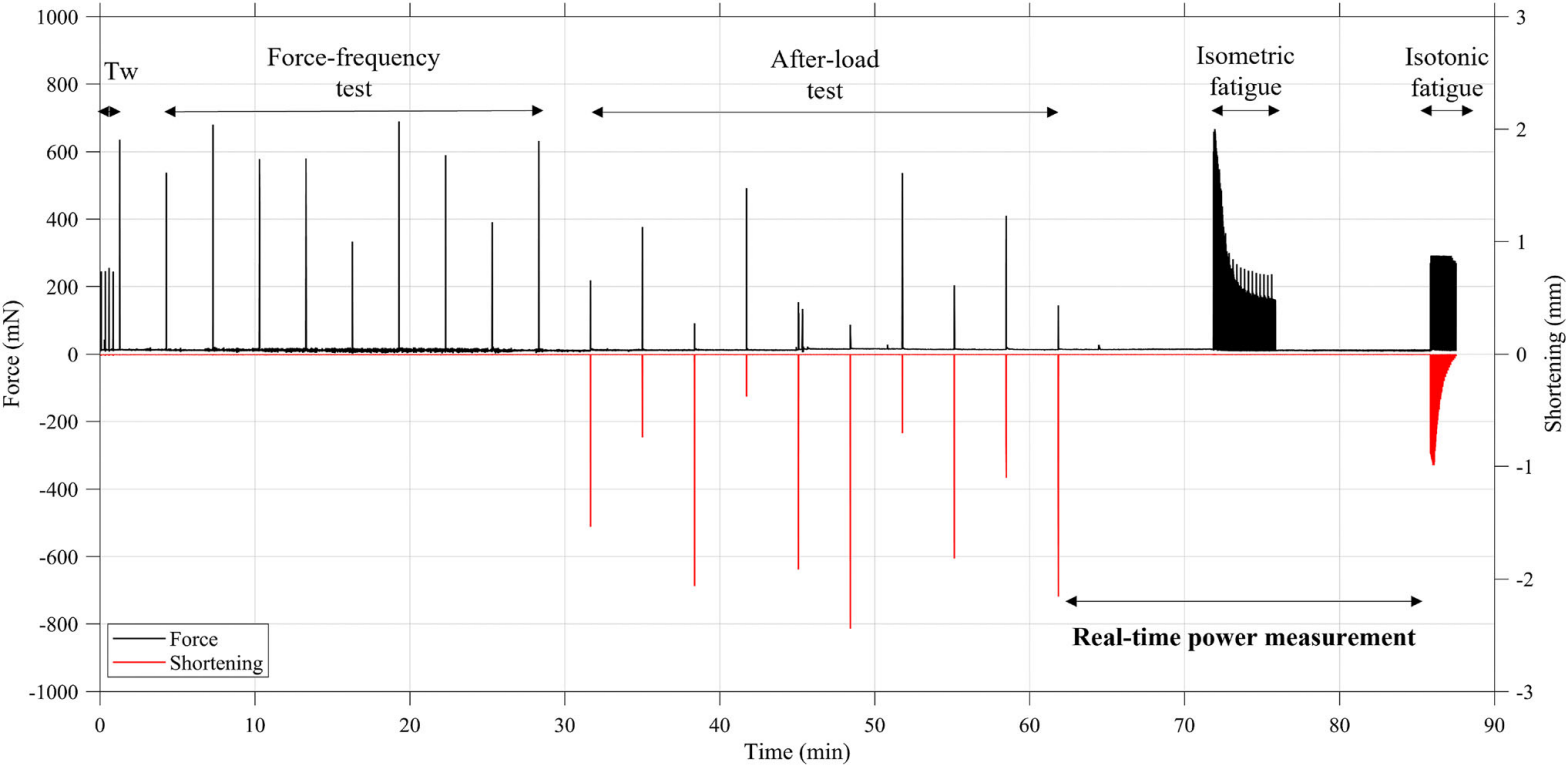
Example of the experimental protocol for the measurement of NMJ functionality: force (black) and shortening (red) values evoked for one 5-month-old WT TA muscle, stimulated alternatively directly and indirectly. For all the tested muscles the force measured at the beginning of the isometric fatigue phase was at least 90% of the tetanic force measured during the force-frequency phase.

To fully characterize the NMJ in isotonic conditions, we developed a continuous extensive testing protocol to measure the alterations of synaptic transmission between TA muscle and sciatic nerve. The protocol was constituted of five different phases: (1) twitch test in isometric conditions, (2) force-frequency isometric test, (3) after-load test in isotonic conditions, (4) isometric fatigue paradigm and (5) isotonic fatigue test. Figure 2 shows an example of the entire stimulation protocol with force and shortening values measured for a wild-type TA muscle.

**Figure 3:**
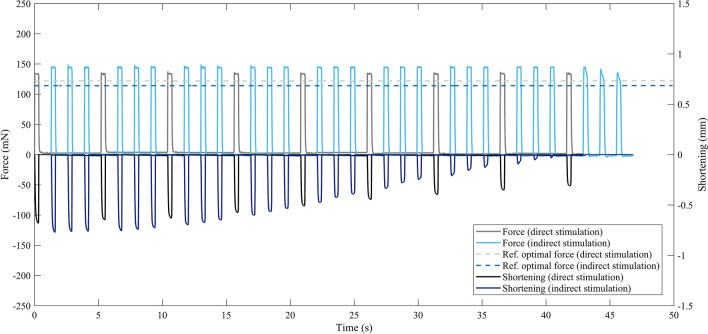
Example of the isotonic fatigue protocol. Direct stimulation: *F*_exp_ = 133.59 mN (36.4% of *F*_Tet_); indirect stimulation: *F*_exp_= 147.16 mN (42.8% of *F*_Tet_). The fatigue test ended when the muscle was not able to shorten following the indirect stimulation.

The muscle was initially held isometric and stimulated alternatively with four 1 ms single pulses, two delivered directly and two through the nerve with a rest period of 15 s between each one. Time to peak (TTP), half relaxation time (1/2RT), maximum value of force derivative (dF/dt) and Twitch Force (F_Tw_) were measured from the direct and indirect twitch responses to characterize the contractile kinetics of the tested muscle. After a resting period of 25 s, the muscle was stimulated with a series of 0.3 s pulse trains at a frequency ranging from 30 to 150 Hz to compute the force-frequency curves for direct and indirect stimulations. In detail, the TA was subjected to the following sequence of stimulations: N90 (indirect stimulation through the nerve at 90 Hz), M60 (direct stimulation on muscle membrane at 60 Hz), N150, M120, N60, M30, N120, M90, N30, M150. In particular, this random order of frequency of pulse train was chosen with the aim of avoiding muscle adaptation to increasing or decreasing values of frequency. A rest time of 180 s was imposed before each stimulation to allow the muscle to completely recover its contractile capability. The third phase of the protocol started after a resting interval of 200 s and consisted in the application of the after-load technique in isotonic conditions for the measurement of muscle maximum power. A series of 0.3 s pulse trains at a frequency of 120 Hz was applied to the muscle through the nerve and on its membrane, simultaneously controlling the load that the tibialis had to lift. The resistive load values were in the range of 10 to 80% of maximum tetanic force, both for direct and indirect stimulations, in a random order to avoid muscle adaptation to increasing or decreasing loads. In detail, they were delivered in the following sequence: N30 (nerve stimulation at 30% of maximum tetanic force), M60 (muscle membrane stimulation at 60% of maximum tetanic force), N10, M80, N20, M10, N80, M30, N60, M20. It is worth noting that the resting times imposed before each stimulation, and in particular during this heavy stimulating paradigm, were chosen to guarantee that the muscle was always able to generate a maximum force comparable to the unfatigued one. Indeed, during this phase of the protocol, a resting period of 200 s was set before each stimulation. Hence, at the end of this phase, the software was programmed to compute the Hill’s curve,^14^ the power-force curve and, therefore, the experimental optimal force (*F*_exp_), namely the force at which the muscle was able to generate its maximum power. This procedure was carried on in parallel with the subsequent stimulation phases, to identify the proper force values to be used in the isotonic fatigue test for direct and indirect stimulations. Indeed, after a resting period of 10 min, in the fourth part of the protocol the muscle was subjected to a fatigue test in isometric conditions, aimed at stressing the neuromuscular junction functionality.^17^ In particular, the muscle was stimulated on its membrane with one 0.3 s pulse train at 120 Hz, followed by fourteen 0.3 s pulse trains at 120 Hz delivered through the nerve, interspersed with 0.7 s of rest. This sequence was repeated 16 times.^22^ In this part of the protocol, the Neurotransmission Failure (NF) and the Intratrain Fatigue (IF) were measured to evaluate the fatigue of synaptic transmission in isometric conditions.^1,22,27^ The Neurotransmission Failure was computed as

1 $$NF= \frac{F-MF}{1-MF}\%,$$

where the force reductions in indirect ($F$) and direct ($MF$) stimulations were computed as the difference between maximum forces developed by the muscle at the first stimulation and at the others, normalized on the tetanic force generated at the first stimulation. The Intratrain Fatigue was computed as the ratio between the force generated by the muscle at the end of the pulse train and the maximum force generated during the same pulse train, in percent. As for the indirect stimulation, NF and IF values were computed on the first pulse train immediately after the direct stimulation. After a resting interval of 10 min, the experimental protocol ended with the fatigue test in isotonic conditions. To stress the junction and to determine a parameter able to represent the differences in control and pathological NMJ, we performed a series of preliminary experiments by varying the number of pulse trains delivered through the sciatic nerve and the resting period after each isotonic phase. These preliminary tests suggested that the optimal isotonic fatigue protocol was characterized by one 0.3 s pulse train delivered on TA membrane, followed by three 0.3 s pulse trains applied through the sciatic nerve, with a resting period of 1 s. Indeed, in all the tests we conducted, the fatigue of synaptic transmission in indirect stimulation occurred earlier than in direct one. The frequency of the pulse train was 120 Hz for direct and indirect stimulations, and the muscle was allowed to shorten against a load equal to the experimental optimal force (F_exp_) computed once the after-load phase was concluded.^9^ An example of isotonic fatigue test for a wild-type TA is reported in Fig. 3. As can be seen, the experimental optimal force (F_exp_) was higher than reference value (F_ref_), equal to the 33% of the tetanic force, both for direct and indirect stimulations. The fatigue test ended when the muscle was no longer able to shorten against the resistive load, and the fatigue time (T_Fat_) was measured as the time necessary to fatigue the specimen. As previously said, this condition always occurred when stimulating the muscle through the nerve, as in the example of Fig. 3.

To characterize the NMJ transmission during the isotonic fatigue protocol, we devised an innovative parameter similarly to what has been done for the isometric fatigue,^1^ the *Isotonic Neurotransmission Failure (INF)*:

2 $$INF= \frac{NS-MS}{1-MS}\%$$

where *NS* and *MS* are the shortening decrease after nerve and muscle stimulation and were computed as the difference in maximum shortening between the first stimulation and the subsequent ones, normalized on the maximum shortening generated at the first stimulation. As regards the nerve stimulation, the *INF* was computed on the first pulse train immediately after the direct stimulation on muscle membrane. As proof of concept of the adequacy of this parameter, we measured it for TA muscles excised from SOD1^G93A^ mouse model at the end-stage of the disease in comparison to age-matched WT specimens. *INF* was computed at two different stages of the isotonic fatigue phase: in the middle (50%) and shortly before (80%) the end. Indeed, since the isotonic fatigue test ended when the muscle was no longer able to lift the resistive load, it was not possible to measure the *INF* at the end of the test (*INF *= 100%). To this, to increase INF accuracy it was necessary to determine in advance the timepoint at which it must be evaluated.

The total power and work generated by the muscle during the fatigue test were computed as the sum of the product of the constant load for the highest shortening velocity and displacement during each direct and indirect shortening, respectively. Normalized power and work were obtained dividing maximum power and work by muscle mass, respectively. At the end of the test, the mouse was sacrificed by cervical dislocation to minimize suffering. TA muscle length and mass were measured for data normalization through an analog caliper, with an accuracy of 0.05 mm, and a Pioneer precision scale (Ohaus, Parsippany, NJ), with an accuracy of 0.1 mg. Muscle cross sectional area (CSA) was estimated as previously reported.^9^

### Real-Time Measurements of Muscle Maximum Power

The data acquisition frequency was set at 20 kHz for single pulse stimulation and 1 kHz for all pulse train stimulations. Muscle tetanic force (*F*_tet_) was determined from the force-frequency curve, for direct and indirect stimulations, and it was then employed to obtain the values of resistive loads to be imposed during the after-load test in isotonic conditions. Based on our previous work,^9^ we developed a sub-VI (LabVIEW 2019, NI) for the real-time measurement of the maximum power generated by the muscle when stimulated on its membrane and through the nerve, to be run in parallel of the main program. The Hill’s curve,^14^ representing the relationship between muscle force (*F*) and shortening velocity (*v*), was computed interpolating the experimental data by using the ‘Nonlinear Curve Fit’ VI (LabVIEW 2019, NI), on the hyperbolic equation:

3 $$\left(F+a\right)*\left(v+b\right)=c$$

where *a*, *b*, *c* are positive constant values. In addition to the interpolation on the 5 experimental points, the curve was forced to pass through the point (*v* = 0; *F* = *F*_tet_). Then the program estimated the power delivered by the muscle by multiplying the resistive loads to shortening velocity values over the entire range of forces. Finally, the experimental optimal force (*F*_exp_) was computed as the force value corresponding to the maximum power, both for direct and indirect stimulations.

### Statistical Analysis

Differences in all the parameters computed for direct and indirect stimulations in WT animals were evaluated with unpaired Student’s t-test, as well as differences in the parameters computed for WT and SOD1^G93A^ mice during fatigue test, namely *INF* at 50%, *INF* at 80% and *T*_Fat_. Statistical analysis was performed with GraphPad Prism 6.0 and differences were considered significantly when p-value was lower than 0.05. Values are expressed as mean ± SD.

## Results

### Isometric Test Parameters

Table 1 summarizes all the parameters measured during the isometric phase, i.e., the parameters measured during twitch test, fused and unfused tetanic stimulations and isometric fatigue protocol. For all these parameters no statistically significant differences were obtained between the values measured when the TA muscles were stimulated directly and through the nerve. In addition, the coefficient of variation (CV) values measured for the direct and indirect stimulations were in good accordance with each other.

**Table 1:** Isometric test parameters.

	WT muscle	WT nerve
TTP (ms)	26.61 ± 7.72	25.91 ± 5.89
1/2RT (ms)	23.51 ± 8.54	24.59 ± 6.88
d*F*/d*t* (mN/ms)	10.70 ± 3.57	11.23 ± 3.80
*F*_Tw_ (mN)	156.20 ± 52.05	161.70 ± 50.05
*F*_Tw_/CSA (mN/mm^2^)	26.18 ± 9.51	26.93 ± 9.64
*F*_Tet_ (mN)	465.90 ± 125.40	485.90 ± 126.30
*F*_Tet_/CSA (mN/mm^2^)	77.64 ± 27.99	79.88 ± 22.18
IF (%)	85.03 ± 10.46	75.43 ± 11.98

Kinetics contractile parameters (TTP, 1/2 RT, d*F*/d*t*), Twitch force (*F*_Tw_), specific Twitch force (*F*_Tw_/CSA), tetanic force (*F*_Tet_), specific tetanic force (*F*_Tet_/CSA) and Intratrain Fatigue (IF) of tibialis anterior specimens in WT mice, stimulated on the muscle and through the nerve. Values are mean ± SD. *n* = 8

As for the NF, we measured a maximum average value of 41.21 ± 10.21% (*n* = 8), with a coefficient of variation in high agreement with the literature. This entire data set confirmed the quality of the proposed protocol for the isometric measurements.

### Muscle Maximum Power and Experimental Optimal Force

**Figure 4:**
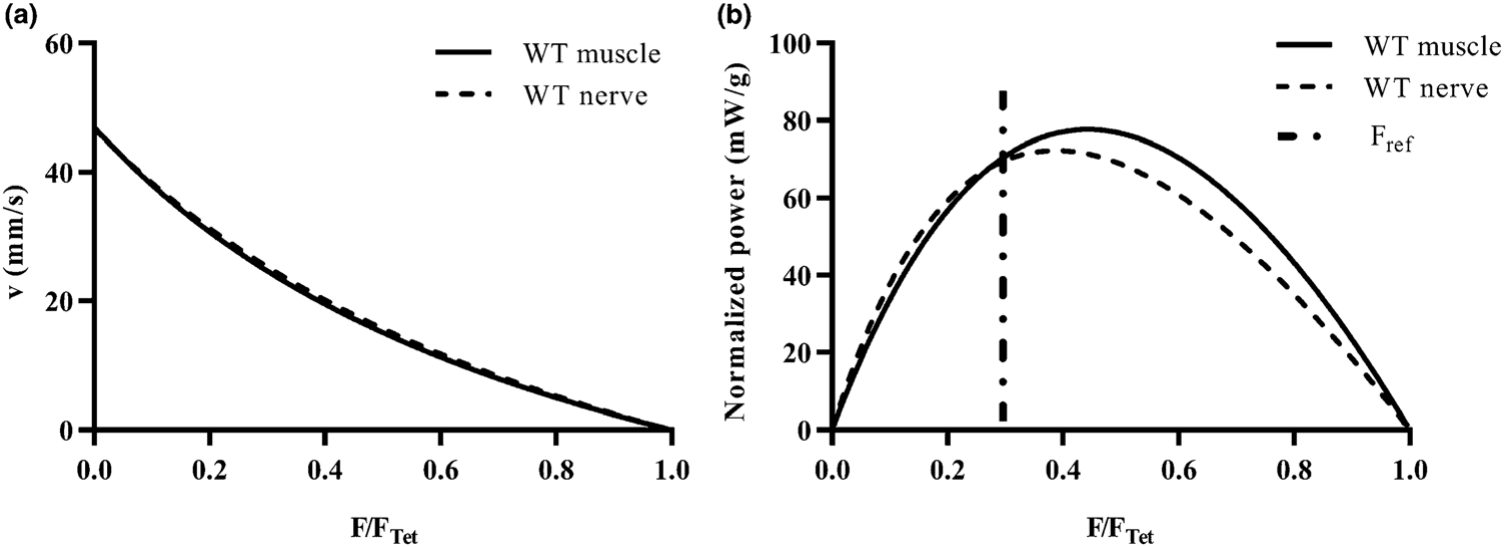
Example of Hill’s curves (*F*/*F*_Tet_ – *v*) (a) and normalized power (*F*/*F*_Tet_ – P/muscle mass) curves (b) of one WT tibialis anterior muscle, for the direct (muscle) and indirect (nerve) stimulations.

Figure 4 shows an example of the Hill’s curves and normalized power curves for a wild-type TA muscle. In particular, Fig. 4(a) points out that the Hill’s curve computed for the nerve stimulation is almost coincident to the one obtained for the direct stimulation. As a consequence, the maximum shortening velocity calculated through the nerve stimulation (47.00 mm/s) was highly close to the one obtained for the direct stimulation (46.82 mm/s). On the other hand, Fig. 4(b) shows that the maximum power was generated by the muscle at a force level higher than the reference one, equal to one-third of the corresponding tetanic force (*F*_ref_ = 33% of *F*_Tet_), both for direct and indirect stimulations.

Table 2 shows the isotonic parameters obtained through the application of the after-load technique, namely maximum power (*P*_max_), normalized maximum power (*P*_max_/muscle mass), maximum shortening velocity (*v*_max_), experimental (*F*_exp_) and reference (*F*_ref_) optimal force, for WT tibialis anterior muscles. For all the measured parameters, the coefficient of variation obtained stimulating the specimen through the nerve, was in high accordance with the corresponding CV obtained for direct muscle stimulation, thus supporting the consistency of the proposed technique for isotonic evaluation of NMJ functionality. To evaluate the efficacy of the real-time measurement of the maximum muscle power, and therefore the consequent determination of the experimental optimal force, we also reported the measurement of the reference value of the optimal force for each muscle as one-third of the tetanic force, both for muscle and nerve stimulations. Of note, in all the experiments conducted in this work, the force value corresponding to the maximum power generated by the TA muscle, stimulated directly and indirectly, was higher than the theoretical one. On average, the relative errors computed as the difference between the theoretical and experimental optimal force values normalized to the theoretical one, resulted equal to 18.21 and 24.25%, for the direct and indirect stimulations respectively.

**Table 2:** After-load test parameters.

	WT muscle	WT nerve
*P*_max_ (mW)	4.24 ± 2.08	5.21 ± 2.60
*P*_max_/m (mW/g)	84.31 ± 37.10	101.70 ± 39.56
*v*_max_ (mm/s)	55.45 ± 15.58	59.40 ± 18.16
*F*_exp_ (mN)	184.40 ± 48.66	202.40 ± 43.96
*F*_ref_ (mN)	156.00 ± 41.69	162.90 ± 42.25

Maximum power (*P*_max_), normalized maximum power (*P*_max_/*m*), maximum shortening velocity (*v*_max_), experimental optimal force (*F*_exp_) and reference optimal force (*F*_ref_) of tibialis anterior specimens in WT mice, measured through the after-load technique for muscle and nerve stimulations. Values are mean ± SD. *n* = 8

### Isotonic Fatigue Test Parameters

Table 3 shows total maximum power (*P*_tot_), normalized total maximum power (*P*_tot_/muscle mass), total maximum work (*W*_tot_) and normalized total maximum work (W_tot_/muscle mass) measured during the isotonic fatigue test for WT tibialis anterior muscles. Also for these parameters, no significant differences were reported between the values measured when stimulating the muscle through the nerve and on its membrane, as well as the CV computed in the two cases was in high agreement.

**Table 3:** Isotonic fatigue test parameters.

	WT muscle	WT nerve
*P*_tot_ (mW)	25.55 ± 12.22	29.32 ± 11.57
*P*_tot_/*m* (mW/g)	477.48 ± 170.68	553.64 ± 163.24
*W*_tot_ (mJ)	1.19 ± 0.54	1.41 ± 0.67
*W*_tot_/*m* (mJ/g)	22.32 ± 7.80	26.65 ± 10.55

Total maximum power (*P*_tot_), normalized total maximum power (*P*_tot_/*m*), total maximum work (*W*_tot_) and normalized total maximum work (*W*_tot_/*m*) of tibialis anterior specimens in WT mice, computed during the fatigue test, for muscle and nerve stimulations. Values are mean ± SD. *n* = 8

### Synaptic Transmission During Isotonic Fatigue Test

**Figure 5:**
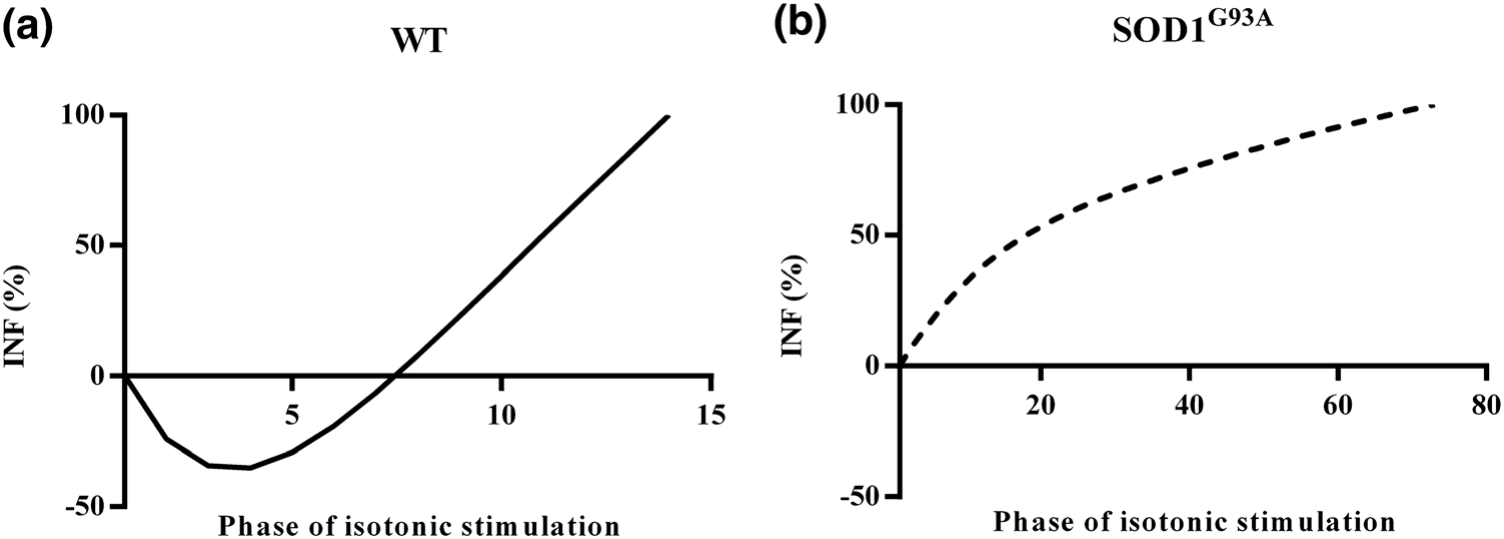
Example of Isotonic Neurotransmission Failure curves for TA muscles in wild-type (a) and SOD1^G93A^ (b) mice.

Figure 5 shows an example of the *Isotonic Neurotransmission Failure (INF)* curves computed for one TA excised from a WT and a SOD1^G93A^ mouse. Figure clearly shows that the trend of the *INF* curves was highly different between control and transgenic muscles, pointing out a different response to the proposed isotonic fatigue test. The *INF* curves reported in Fig. 5 are representative of all the tested WT and SOD1^G93A^ muscles both in terms of trend and higher fatigue time. As expected, the WT muscle fatigued earlier than the SOD1^G93A^ one (14 isotonic stimulations versus 73 stimulations in the example).

**Figure 6:**
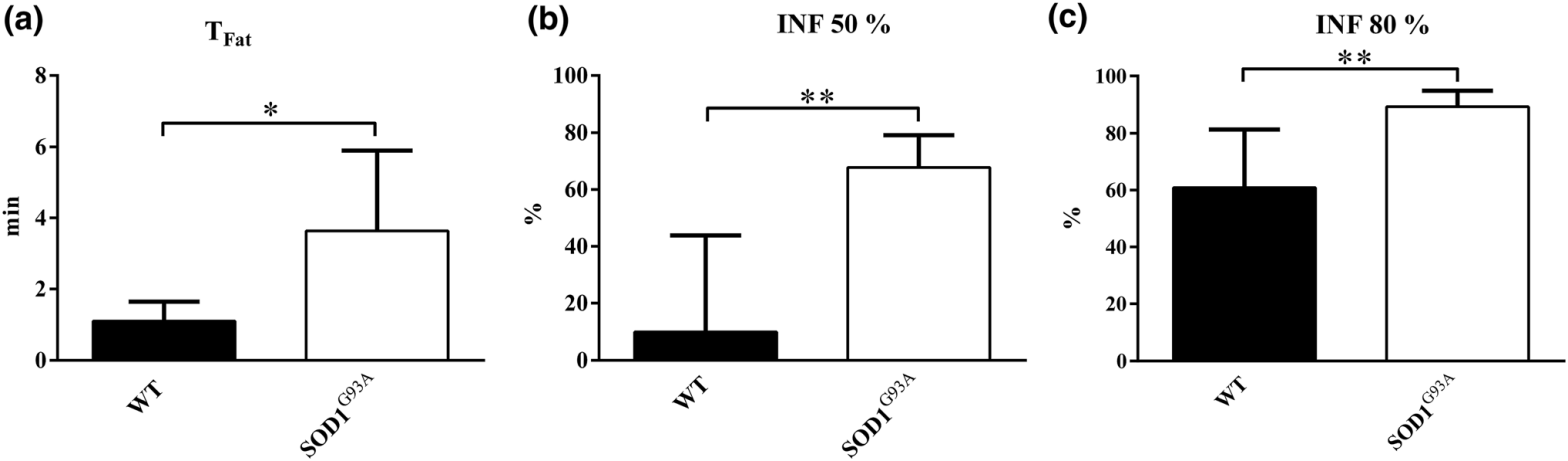
Fatigue time (T_Fat_) (a) and Isotonic Neurotransmission Failure (INF) at 50% (b) and 80% (c) of isotonic fatigue test. Values are mean ± SD. *n* = 7 for WT, *n* = 5 for SOD1^G93A^. *p-value < 0.05. **p-value < 0.01.

Figure 6 shows the fatigue time (*T*_Fat_) and the *Isotonic Neurotransmission Failure* computed at 50 and 80% of the duration of the isotonic fatigue test. As shown in Fig. 6(a), the fatigue time in transgenic muscles was significantly higher than the one measured in control ones. *INF* values at 50 and 80% reported a significant increase of about 300 and 50%, on average, in synaptic transmission for transgenic mice when compared to control ones. These results confirmed that the parameter we proposed was properly capable of assessing an impairment in the synaptic transmission during isotonic fatigue test.

## Discussion

The aim of this work was to device a novel experimental technique for the measurement of neuromuscular junction (NMJ) functionality of mice models in isotonic conditions. Indeed, even if the study of the altered communication between muscle and nerve is crucial in several neurodegenerative diseases,^5,10,24^ to date, NMJ functionality has been measured only in isometric conditions. To this aim, we took advantage of the *in-situ* testing technique, studied a proper placing of two pairs of wire electrodes, one for nerve and one for muscle stimulation, developed an extensive testing protocol for the measurement of muscle and NMJ functionality in isometric and isotonic conditions, and proposed a novel parameter to properly capture the impairment in neurotransmission during the isotonic fatigue test. Of note, to increase the measurement accuracy of the parameters related to isotonic fatigue, we also devised a new approach based on the use of the experimental optimal force, i.e. the force at which the muscle develops its maximum power, instead of using the standard value of optimal force proposed in the literature (1/3 of the maximum force).^9^As expected, for all the isometric parameters measured when stimulating the muscle through the nerve and on the membrane, no statistically significant difference was reported. The values for the single experiments differed always less than 10% for each parameter, a value in high agreement with those reported in the literature for isometric evaluation of NMJ functionality.^7,17,19^ In addition, also the values of the *Neurotransmission Failure*, a parameter that allows evaluating the fatigue of synaptic transmission in isometric conditions, returned a variation around the average value highly consistent with the literature, confirming that the electrode positioning here employed did not alter the quality of the measurements in isometric conditions.^11^

As for the isotonic measurements, our results showed a very good agreement between the Hills’ curve obtained during the after-load phase for direct and indirect stimulations. Consequently, no significant differences were reported for all the parameters measured in this part of the protocol for nerve and muscle stimulation, indicating a good feasibility of the proposed technique. Moreover, the coefficient of variation measured during the nerve stimulation was also in high accordance with the corresponding value obtained for direct muscle stimulation.

Interestingly, from this part of the protocol we also measured the experimental optimal force and showed that it was higher than the reference optimal one for all the tested specimens, with a relative increase of about 20% on average. Therefore, if the TA muscles were tested to shorten against the reference optimal force during the isotonic fatigue test, they would have been tested in a condition very far from the one allowing them to generate their own maximum power. As a matter of fact, in a previous work^9^ we demonstrated that the use of the experimental optimal force as the resistive load during isotonic fatigue test, leaded to a significant decrease in the variance of all the parameters measured in this phase, as fatigue time (the CV decreased from 61.4 to 18.4%), mechanical power (CV from 69.24 to 33.10%) and work (CV from 57.55 to 24.49%). These results confirmed the need of an optimization of the isotonic fatigue protocols proposed in literature.

As for the novel isotonic test here proposed, the coefficient of variation obtained for the maximum power and work measured stimulating the muscle directly and indirectly were in good agreement among each other, confirming the feasibility of the proposed technique during the isotonic fatigue phase. Interestingly, the CV values here obtained when stimulating the TA muscles on the membrane were also in accordance with the values obtained when testing TA to fatigue in isotonic conditions only with direct membrane stimulation,^9^ confirming that the use of a combined stimulation (direct and indirect) did not affect measurement accuracy.

With the aim of deeply investigating the synaptic transmission in isotonic conditions, we developed a fatigue testing protocol and proposed a novel parameter, named the *Isotonic Neurotransmission Failure*. Experimental results pointed out a different behavior for ASL SOD1^G93A^ muscles and control ones. Indeed, for SOD1^G93A^ TA muscles the shortening decrease occurring during nerve stimulation was higher than that obtained with membrane stimulation from the beginning of the electrical stimulation, while for WT muscles this condition occurred about after half of the fatigue test. At this point, it has to be remarked that this behavior was a direct consequence of the protocol we devised, in which 1 membrane stimulation was followed by 3 nerve stimulations. The rationale behind this protocol was to obtain a test sensitive enough to reveal all the differences occurring in an animal model for which alterations in the synaptic transmission were already reported,^7,8,17^ even if only in isometric conditions, without over-stressing the synaptic transmission in control specimens. An increase in the number of nerve stimulations would have led, for example, to a higher decrease in shortening during indirect stimulations from the very beginning of the test, also for the WT specimens. The fatigue time measured with this protocol was significantly higher in the SOD1^G93A^ samples than in WT ones, in accordance with the progressive failure of the NMJ functionality and the consequent energy management by mitochondria in ALS SOD1^G93A^ mouse model already reported.^26^ Indeed, the *INF* measured at 50% and at 80% of the protocol length resulted significantly higher in SOD1^G93A^ TA, pointing out that, even if these transgenic muscles showed a greater resistance to fatigue, they underwent a bigger impairment in the synaptic transmission in comparison to WT ones. Again, these results confirmed the complexity of the mechanisms behind the resistance to fatigue, as the energy management by mitochondria,^26^ and the need of novel approaches and parameters to improve the knowledge of this altered behavior.

In conclusion, we devised a novel experimental technique and testing protocol for a deep investigation of NMJ functionality in isometric and, for the first time, isotonic conditions. Of note, the muscle shortening did not alter measurement accuracy, and the entire testing protocol allows measuring a huge variety of parameters. On the other hand, the *Isotonic Neurotransmission Failure* parameter we proposed was properly capable of assessing an impairment in the synaptic transmission during isotonic fatigue test. Finally, even if we took advantage of an ALS animal model as proof-of-concept of *INF* validity, the technique and the testing protocol here proposed may contribute to unravel new mechanisms behind several other neurodegenerative pathologies, as aging and Duchenne Muscular Dystrophy.
